# Coating of TPU-PDMS-TMS on Polycotton Fabrics for Versatile Protection

**DOI:** 10.3390/polym9120660

**Published:** 2017-11-30

**Authors:** Arsheen Moiz, Rajiv Padhye, Xin Wang

**Affiliations:** School of Fashion and Textiles, RMIT University, Melbourne 3056, Australia; s3494882@student.rmit.edu.au (A.M.); rajiv.padhye@rmit.edu.au (R.P.)

**Keywords:** polycotton, superhydrophobic, oil, chemical, comfort

## Abstract

This research aims to develop a non-fluorine based and durable coating technology that brings excellent hydrophobic, oleophobic and aqueous liquid repellent properties to polycotton fabrics (blend ratio 80/20 for cotton/polyester) while maintaining comfort to an acceptable level. A crosslinked network from thermoplastic polyurethane (TPU), polydimethylsiloxane (PDMS) and trimethylated silica (TMS) has been formed on the surface of polycotton fabrics by the conventional padding-knife coating-padding-curing technique. A series of characterizations have been conducted to understand the chemical components, morphology, versatile protection and comfort of the coated fabrics. The TPU-PDMS-TMS (TPT) coated fabrics showed a high hydrophobic surface with a high water contact angle of 142°, and the coating was durable against different cycles of laundering and crocking. The coated fabrics also showed excellent repellency against oils, liquids and chemicals for a long period of time. The coating has affected the air permeability and water vapor permeability together with the moisture management property of the polycotton fabrics, and the thermal resistance of the polycotton fabric has been enhanced at the same time. The coating technology developed can be further applied in protective clothing and functional textiles in different areas including military, mining and outdoor protection gear.

## 1. Introduction

Coating is a very effective technology that brings extra functions to the substrate materials. A classic example of this is the coating for superhydrophobicity towards self-cleaning textiles, in which the superhydrophobic surface is measured by a water contact angle of >150° [1]. Usually for self-cleaning textiles, the apparent contact angle is very high (150–170°) and the angle of hysteresis is very low for the water droplets to roll off from the surface of the fabrics. Similar to hydrophobicity, oleophobicity has been developed to bring resistance against oils to fabrics. Chemical resistance is another important factor for protective clothing, and it is very effective in surface decontamination for military uniforms, mining clothing and outdoor sportswear [2]. When the surface of textiles is not penetrated by water, oils, and liquids chemicals (such as acids, base and solvents), it is called omniphobic surface. The omniphobic surface of clothing is helpful in protecting skin against hazardous liquid chemicals, industrial chemicals, petroleum oils, lubricants and bacterial viruses. Versatile protection from the developed omniphobic surface of textiles is crucial for customers who are facing threats from either environment or working places.

Comfort is another important factor for developing protective clothing, as the widely applied coatings usually affect the thermal and moisture behavior of the fabrics (especially their breathability which will severely deteriorate due to the blocking of pores in the fabrics). Polytetrafluoroethylene (PTFE) has been used to develop protective textiles under the brand Gore-Tex^®^. With a water contact angle of 110°, Gore-Tex has the properties of waterproof, breathability and windproof that make it applicable in various areas. The success of Gore-Tex indicates that a balance between protection and comfort is the key in developing protective textiles. Besides, fluoro-based polymers or compounds have been widely applied to form superhydrophobic surfaces [3,4]. However, toxic byproducts, such as perfluorooctanoic acids (PFOA) and perfluorooctane sulphonates (PFOS), are usually produced in the synthesis of C8-based fluorocarbon resins, and removal of them would result in high costs. Replacement of fluoro-based polymers for protective coating on fabrics is of great interests to textile industry [5]. Eco-friendly polymers have been developed to be the ideal replacement in this regard [6]. Among them, polyurethane and silicone together with its derivative compounds are promising in developing superhydrophobic surfaces on fabrics towards versatile protective clothing [7,8]. Polyurethane (PU) has been widely applied as coating materials for breathable but wind and water proof membranes [9], and as flame retardant materials for functional textiles [10,11]. The thermoplastic polyurethane (TPU) based materials have found to be suitable for protective textiles such as facemasks which resist chemical warfare agents (CWA) [12]. Microporous PU membrane with self-decontaminating agent (polyoxometalate) were used for decontaminating the G-agent and distilled mustard (HD) surrogates [13]. With good membrane forming ability, polyurethane itself can provide protection to textiles. However, the surface of polyurethane would need further functionalization to introduce value-added protection mechanisms. A laminated coating of microporous PU membrane with polytetrafluoroethylene (PTFE) was used to form water and vapor resistant fabrics for sportswear [14]. Waterproof-breathable PU nanofibers coating has been used as high performance protective clothing for sportswear industry [15,16]. As an environmental friendly polymer, PU has been modified by 5,5-dimethylhydantoin to form multifunctional and self-decontaminating biocidal surface to remove the gram positive and negative bacteria [17].

In order to develop versatile protection against different agents, PU has been combined with other protection mechanisms to enhance the protection capacity [18]. Recent research on polydimethylsiloxane (PDMS)-based silica surface or silica based polymer surface have shown oil-water separation and versatile protection [19,20]. Waterborne polyurethane was combined with PDMS-TMS to produce a hydrophobic surface with low surface tension and high resistance to water, oil and chemicals [21]. The chemical resistance to methanol, acetone and *iso*-propyl alcohol requires a low surface tension of around 25 dynes/cm. Superoleophobic surface usually has a surface tension of less than 20 dynes/cm [22], while superhydrophobic surface usually has a high surface tension of 72 dynes/cm [23]. It would be very challenging to achieve a superomniphobic surface that is resistant to water, oil and chemicals in terms of reaching a compromised surface tension of the coated surface. A combination of waterborne polyurethane (WPU) crosslinked with PDMS and trimethylated silica (TMS) has been developed for this purpose to resist different agents including water, oil and chemicals [21]. However, due to the existence of the uniform membrane of WPU, the comfort, handle and flexibility of the coated cotton fabrics have been severely affected. It is a big challenge to develop versatile protective coating while maintaining proper comfort of fabrics. Changes in the polyurethane, either its chemical composition or its structure, are needed for further development of polyurethane based versatile protective clothing.

A soft, flexible and protective coating that does not compromise much of the breathability of fabric structure is needed to address the challenge. It is thus important to study the substrate materials of polyurethane, and an ideal substrate would facilitate the functional coating while maintain the comfort the as-coated fabrics. This study investigates the coating of thermoplastic polyurethane (TPU) combining with polydimethylsiloxane and trimethylated silica (PT) using a three-step pad-knife-pad coating on polycotton (PC) fabrics. A three-layered hierarchical structure based on the complex modification of TPU by PT has been obtained to form crosslinking reaction, in which the polydimethylsiloxane reacts with the amino groups of the polyurethane to form Si–OCH_3_ compounds. The TPU-PDMS-TMS coating has shown higher hydrophobicity with better durability, more flexible handle and better thermophysiological comfort compared to the WPU-PDMS-TMS coating. This study will benefit the development of alternative coating processes to replace the fluoropolymers based coating technology.

## 2. Experimental

### 2.1. Materials

Blended fabrics polycotton (150 g/m^2^, plain) were purchased from Bruck Textiles, Abbotsford, Australia. The fabrics were fabricated from cotton and polyester fibers with a blend ratio of 80/20 for cotton/polyester, respectively. Tubicoat Fix ICB conc was provided by CHT Australia Pty Ltd., Dandenong South, Australia. Thermoplastic polyurethane (TPU, Texalan-598-A, in pellets form) were purchased from Pacific Urethanes Pty Ltd., Carrum Downs, Australia. Xiameter FBL-0563 (15–35% PDMS, 15–35% TMS) was sourced from Dow Corning Pty Ltd., Pennant Hills, Australia. Hexadecane and *n*-decane were purchased from Sigma Aldrich Pty Ltd., Castle Hill, Australia. *N*-heptane (HPLC grade) was purchased from RCL LAB SCAN Limited, Australia. *Iso*-propyl alcohol (Ana-R) was purchased from BDH Limited, Dorset, UK. *N*,*N*-dimethylformamide (DMF) was purchase from Merck KGaA, Darmstadt, Germany.

### 2.2. Methods

The polycotton fabric was wetted by 0.01% (*v*/*v*) Triton X-100 solution in water for 0.5 h and scoured by caustic soda at 90 °C for 1 h. The fabric was rinsed with warm water and then with cold water, followed by an overnight hanging dry. 

**Figure 1:**
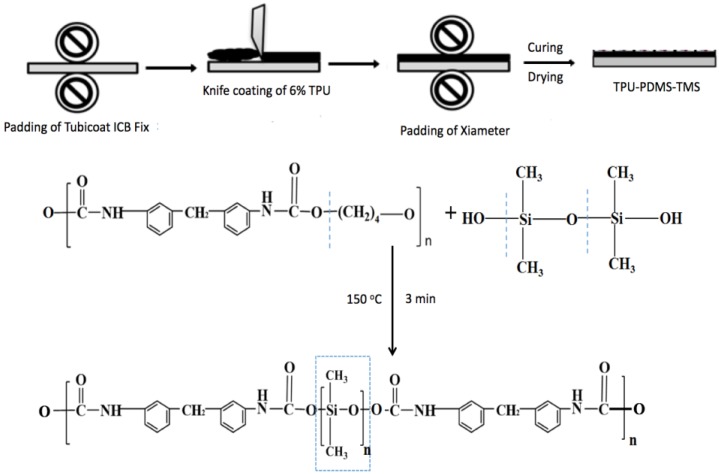
Schematics of pad-knife-pad coating of thermoplastic polyurethane (TPU)-polydimethylsiloxane (PDMS)-trimethylated silica (TMS) (TPT) on polycotton fabrics and the crosslinking mechanism.

The polycotton fabric was then subject to a three-step coating process as illustrated in Figure 1. Firstly, an Ernst Benz Pad was used to perform ICB Fix padding for three passages. The solution in the pad contained 30 mL distilled water with 0.15 g ICB Fix dissolved in it. The fabric was first dipped into the solution to reach thorough impregnation. The fabric with solution impregnated was then passed between the two rollers of the pad to squeeze out air and to force the solution into the fabric. The excess of the solution was sent back along the fabric to the solution bath at the same time. After this *dip-nip* process, the sample was dried at 60 °C for 30 min and the percentage of the total pickup on the fabric was calculated.

Secondly, the padded fabric was coated with 6% TPU paste (prepared by dissolving TPU pellets in DMF) by a knife edge rolling over method. In the coating process, the fabric was mounted on the frame of the coater (Werner Mathis AG, Oberhasli, Switzerland), and the knife was set on the surface of the fabric with a given distance to determine the thickness of the coating. A certain amount of TPU paste was poured in front of the edge of the knife, and the knife was then moving slowly on the surface of the fabric to spread the TPU paste evenly. The thickness of the coating can be controlled by adjusting the distance between the knife edge and the fabric. The coated sample was then dried at 60 °C for 30 min. Thickness of TPU coating was calculated on a thickness tester (British Indicators Ltd., St. Albans, UK) according to the AS 4878.4-2001 method.

Thirdly, the padded and knife coated fabric (6% TPU) was further padded with a PDMS-TMS solution. The PT solution was prepared by diluting the Xiameter FBL-0563 into *n*-heptane with the concentration of 2, 4, 6 and 8%, respectively. After padding, the sample was dried in a lab oven (Electrolux) at 60 °C for 30 min. The sample was then placed in a curing unit (W. Mathis AG, Oberhasli, Switzerland) at 150 °C for 3 min. The three-layered functional coating was then formed on the polycotton fabric based on the crosslinking network of TPU-PDMS-TMS (TPT).

### 2.3. Measurements and Characterizations

#### 2.3.1. Fourier Transform Infrared Spectroscopy (FTIR)

FTIR spectra were obtained from an attenuated total reflection infrared spectrophotometer (Perkin Elmer Spotlight 400, Waltham, MA, USA) with a diamond crystal. During the test, a single layer of fabric was placed on the attenuated total reflectance (ATR) crystal and then the pressure clamp was lowered to provide good contact between the sample and crystal. The scanning range of spectra was 4000–650 cm^−1^ with four scans automatically performed in the spectrophotometer for each test. The test was repeated three times from different areas of each sample.

#### 2.3.2. Scanning Electron Microscopy (SEM)

Surface morphology was measured by a field emission scanning electron microscopy (FESEM, Quanta™-200, Hillsboro, Oregon, USA. The instrument was set at a pressure of 0.45 Torrs, a voltage of 10 kV and a working distance of 10 mm at room temperature. The samples were sputter coated with a thin layer of gold particles (IMBROSE, Spi A20015, West Chester, PA, USA) before being loaded on the platform.

#### 2.3.3. Water Contact Angle

Water contact angle was measured on a contact angle system (Data physics, OCA20, Filderstadt, Germany) at room temperature. A droplet of ultra-pure water (5 μL) from a Milli-Q (Billerica, MA, USA) filtration system was used to evaluate its contact angle on the fabrics. A fixed needle was mounted above the tilting table (on which the fabric sample was placed) with a distance of 10 mm. A syringe was placed so that a droplet from the needle tip can drop onto the surface of the tested fabric. Pictures were taken immediately after the droplet was placed on the fabric surface, and the water contact angle value was calculated by the Sessile Drop Method. The contact angle values were averaged from the results of six replicates. Each replicate was measured five times and each reading was taken from different areas on the fabric.

#### 2.3.4. Water Repellency

The water repellency test was performed in accordance with the American Association of Textile Chemists and Colorists (AATCC) standard 22:2010. Before testing, the coated fabrics were conditioned at 20 ± 2 °C and 65 ± 2% RH for 4 h. The sample with the size of 180 mm × 180 mm was mounted between the test hoops and then 250 mL of distilled water was sprayed onto its surface for over 30 s. The sample was then spray rated using the international standard chart.

#### 2.3.5. Oil Repellency

The oil repellency test was performed in accordance with the standard AATCC 118:2013. Three fabric specimens were cut into the size of 2 mm × 2 mm and were placed in petri-dishes. Three droplets of an oil (10.0 mL each) were placed on the three specimens, respectively. The oil droplets on each fabric were observed from an angle of 45° after 300 s. Six types of oil, namely castor oil, vegetable oil, paraffin oil, *n*-hexadecane, *n*-decane and *n*-heptane, were used to repeat the test. The time for the oil to be absorbed into the fabric was recorded, otherwise 300 s was recorded. In order to test the oil repellency of coated fabrics under tensions, the oil repellency test was repeated under the tension of 1, 3 and 5 numeral pressure (NP), respectively.

#### 2.3.6. Aqueous Liquid Repellency

Aqueous liquid repellency test was performed in accordance with the standard AATCC 193:2012. Three fabric specimens were cut into 2 mm × 2 mm and were placed in petri-dishes. Three droplets of the aqueous liquid (*iso*-propyl alcohol, 10 mL each) were placed on the three specimens, respectively. Four types of aqueous liquids with different water and *iso*-propyl alcohol ratios, namely 98/2 water/alcohol (shopping pink), 90/10 water/alcohol (orange), 80/20 water/alcohol (blue) and 98/2 water/alcohol (yellow), were used to repeat the test. Photos of the droplets on the fabric were taken from an angle of 45° after 10 s and 300 s, respectively. Similarly, the aqueous liquid repellency test was repeated under the tension of 1, 3 and 5 NP, respectively.

#### 2.3.7. Chemical Resistance

The chemical resistance was measured by the same method as that for the oil and aqueous liquid repellency test. Three fabric specimens with the size of 2 mm × 2 mm were placed in petri-dishes, and three droplets of the chemical (10.0 mL each) were placed on these specimens. Photos of the droplets on the fabric were taken from an angle of 45° after 10 s and 300 s, respectively. Sixteen types of Chemicals, namely acetic acids (yellow), sulfuric acids (colorless), sodium hydroxide (green), *n*-hexadecane (blue), dimethylformamide (shopping pink) and isopropyl alcohol (red), were used to do the test. Water was also used in the test for comparison.

#### 2.3.8. Air Permeability

Air permeability was measured on an air permeability tester (SDL Atlas Pty Ltd., Kendal, UK) according to the standard AS-2001.2.34: 1990. During the test, the specimen was clamped over an air inlet of the apparatus while air was sucked through it by a pump. The volume of air passing through the fabric was then measured using a flow meter. Ten specimens were tested and the mean air flow (cm^3^/cm^2^/s) was calculated. The diameter of the test area was 20.0 cm and the pressure was 100 kPa.

#### 2.3.9. Laundering Test

Accelerated laundering test was performed in accordance with the AATCC standard 61: 2013(1A). Fabrics were washed in a SDL Atlas launder-O-meter at 40 °C in the presence of 10 steel balls, and the AATCC reference detergent without any optical brightener was used in the laundering. One 45 min washing cycle is approximately equal to five commercial laundering cycles. Each specimen was then rinsed twice in deionized water and then dried in an air circulating oven for 30 min. All samples were conditioned according to ISO 139 at a temperature of 20 ± 2 °C and RH 65 ± 5% prior to the testing.

#### 2.3.10. Crocking Test

A crock meter (Toyoseiki, Tokyo, Japan) was used to determinate the durability of the coated fabrics against rubbing, in accordance to the standard AATCC 08:2013. Samples (130 mm × 50 mm) were prepared in both the warp and weft directions for wet and dry testing (10 cycles), respectively. A crock meter test cloth was used to determine the crocking fastness under a downward finger force of 9 N.

#### 2.3.11. Thermal Resistance

Thermal resistance (*R*_ct_) was measured on the sweating guarded hotplate (SDL Atlas Pty Ltd., Kendal, UK), according to the standard ISO11092: 1993(E). Three specimens (30 cm × 30 cm) were prepared and conditioned at 20 °C and 65% RH for 24 h. The specimen was then placed on the measuring plate for testing. The parameters of the testing were set as follows: measuring unit temperature (*T*_m_) 35 °C, air temperature (*T*_a_) 20 °C, air circulation speed 1 m/s, and RH 65%.

#### 2.3.12. Water Vapor Resistance

Water vapor resistance (*R*_et_) was measured on the sweating guarded hotplate (SDL Atlas Pty Ltd., Kendal, UK), in accordance with the standard ISO11092: 1993(E). The required power was tested to maintain the temperature of measuring plate (35 °C) for 15 min. During the test, the speed of the circulation air was 1 m/s in the chamber. The permeability index (*I*_m_) was calculated from the value of *R*_ct_/*R*_et_ under the pressure of air (K) as a constant. The ranges of air permeability index varied from 0 to 1 to show the permeability of the fabrics.

#### 2.3.13. Moisture Management Property

The moisture management property of fabrics was measured on the SDL Atlas moisture management tester (MMT), in accordance with the standard AATCC-TM-195 (2009). Five specimens (80 cm × 80 cm) were prepared to do the test under the standard condition. A saline solution was penetrating from the top side to the bottom side of the sample in the test, while the wetting time, wetting radius, spreading time, absorption and overall moisture management capacity (OMMC) were reported on the associated computer.

#### 2.3.14. Handle Property

The handle of the fabrics was tested before and after coating, respectively. The test was conducted by an objective evaluation of the feel of the fabrics. Depends upon the sense of touch, the surface friction, stiffness, flexibility, thickness, luster and compress ability can be compared between different fabrics.

## 3. Results and Discussion

### 3.1. Characterizations

The combined coating of TPU and PDMS-TMS results in a crosslinked network on the surface of polycotton fabrics, and the network brings versatile protection to the fabrics. Tubicoat ICB Fix acts as the crosslinking agent that binds the TPU with polycotton fabrics, providing a durable and flexible substrate for forming crosslinked network. The crosslinking between TPU and PDMS-TMS is schematically depicted in Figure 1. The long chain of TPU combines with PDMS-TMS to form crosslinking, providing flexible and soft segments with siloxane groups to exhibit low surface energy on polycotton fabrics [11,21]. The handle test has found that all the coated polycotton fabrics are stiffer, less soft, shiny in luster with lower bending ability than the uncoated polycotton fabrics. Nevertheless, the TPT coated fabrics are softer and more flexible than the previously developed WPU-PDMS-TMS (WPT) coated fabrics, suggesting improved handle property by adopting TPU rather than WPU.

#### 3.1.1. FTIR Spectra

**Figure 2:**
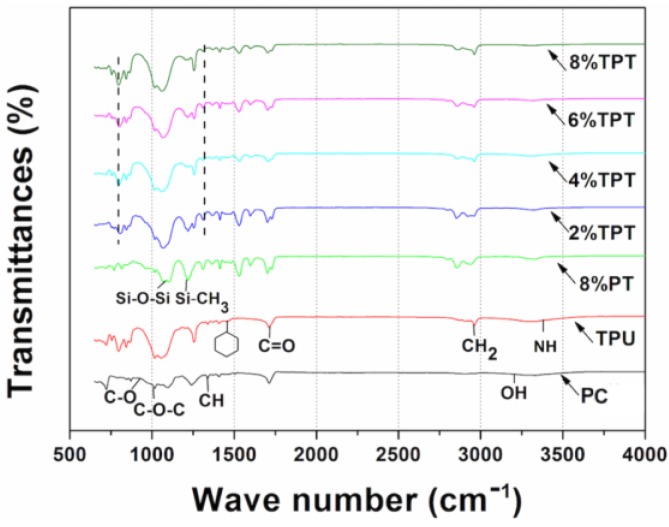
ATR-FTIR spectra of the uncoated polycotton fabric, TPT coated, 8% PT coated and TPT coated with different concentrations of PT.

The chemical components of the coated fabrics were characterized using FTIR-ATR spectroscopy, as shown the spectra in Figure 2. All the functional groups of the samples from the FTIR spectra are listed in Table 1. The spectrum of the uncoated polycotton fabric shows significant bands around 3282, 2917, 1425, 1315, 1054 and 1017 cm^−1^. These bands are attributed to the O–H vibration stretching, the CH_2_ stretching, the CH bending, the asymmetric stretching of C–O–C, and the asymmetric plain stretching of C–O, respectively.

The spectrum of TPU coated polycotton fabric shows characteristic bands of urethane groups, such as NH stretching band at around 3335, 2955 cm^−1^ for CH_2_, and 1728 cm^−1^ for C=O. Besides, there are other relevant bands of the benzene ring structure of TPU including 1597, 1531, 1454, and 1414 cm^−1^. The CH stretching bands can be observed around 848 and 817 cm^−1^, and the NH wagging bands can be identified by the 770, 711, and 663 cm^−1^ bands.

The 8% PT coated polycotton fabric exhibits the symmetric banding of TMS including the bands at 1251 cm^−1^ for Si–CH_3_ and at 1160 cm^−1^ for Si–O–C. In addition, the vibration symmetric stretching bands of Si–O–Si can be observed around 1059, 876, 845 and 770 cm^−1^.

The TPT-coated polycotton fabrics with different PT concentrations show the characteristic bands of both TPU and PT. It is noted that the stretching bands at around 1713 to 749 cm^−1^ are ascribed to the crosslinking of TPU and PDMS-TMS [10,11]. The spectra of TPTs show similar absorption bands, and the characteristic bands of PT are more evident with the increase of the concentration of PT. Specifically, the band intensity of Si–O–Si and Si–CH_3_ increases gradually with the increase of PT concentration.

#### 3.1.2. SEM Photos

**Figure 3:**
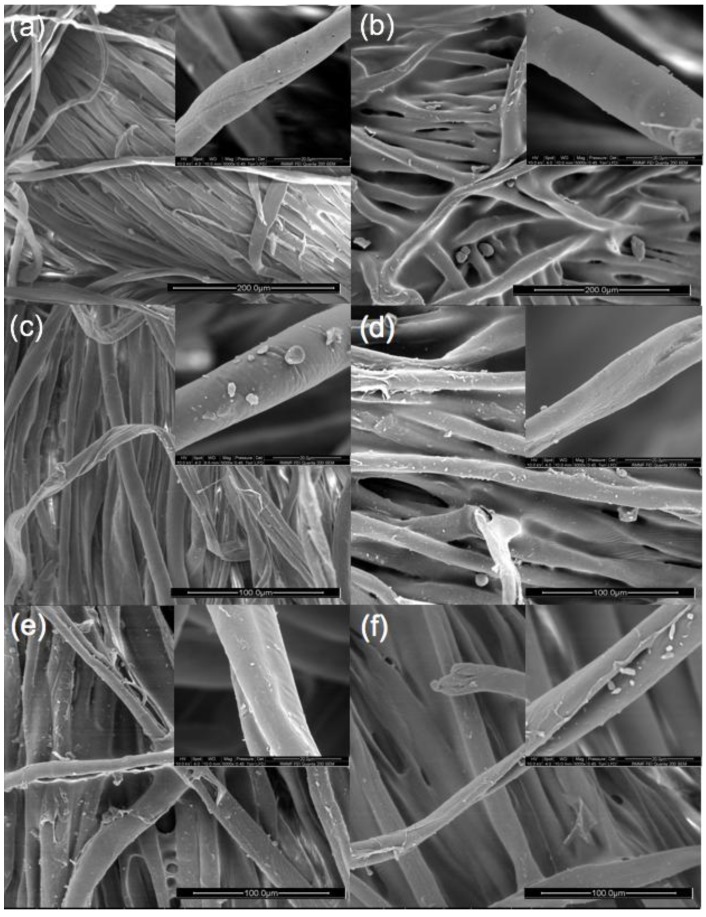
Scanning electron microscopy (SEM) photos of (**a**) uncoated polycotton fabric; (**b**) TPU coated; (**c**) 8% PT coated; (**d**) 2% TPT coated; (**e**) 4% TPT coated; and (**f**) 6% TPT coated (Insets: detailed view with scale bar 20 μm).

The coating has affected the morphology of polycotton fabrics, as indicated in Figure 3. The uncoated polycotton fabric shows a clear fibrous structure with the typical convolutional ribbon profiles of cotton fibers. The inset of Figure 3a shows the clean surface of a cotton fiber from the uncoated polycotton fabric. TPU coating has brought a thin layer to the surface of fibers with neighboring fibers bridging together, resulting in a rather smooth surface morphology as shown in Figure 3b. Detailed view (the inset of Figure 3b) suggests that fiber has been coated successfully. Besides, the fibers have been bridged together as the gaps between neighboring fibers are fed with TPU. Considering single fibers can be seen from the TPU-coated polycotton fabric, TPU coating hasn’t formed a bulky and uniform membrane on the fabric surface. PT padding has hardly affected the morphology of polycotton fabrics but small shiny particles can be observed from the surface of fibers (the inset of Figure 3c). The TPT coating has brought a thin coating layer onto the surface of fibers with particles on it, as shown in Figure 3d–f. More particles can be observed with the increase of the concentration of PT, as seen from the insets. Previous study on waterborne polyurethane coating [21] indicated that a uniform membrane was formed to cover the surface of the cotton fabrics. It can be seen from the SEM photos that the fibrous structure of polycotton fabrics has been partly preserved after TPT coating, and the particles contribute to the surface roughness of the coated polycotton fabric.

### 3.2. Versatile Protection

#### 3.2.1. Water Contact Angle

**Figure 4:**
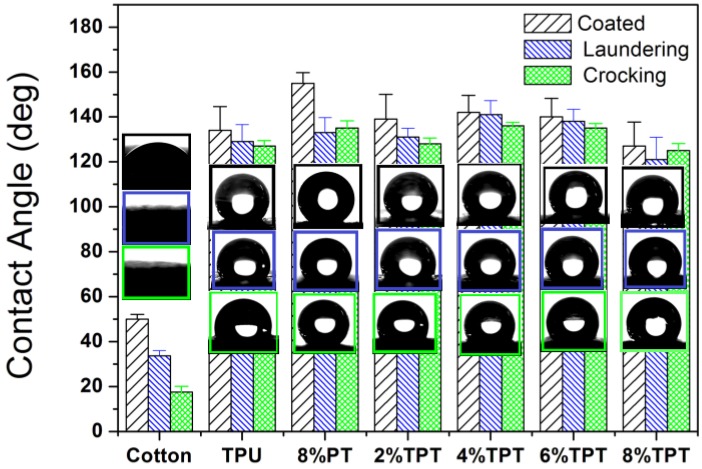
The water contact angles of the uncoated polycotton fabric, TPU coated, 8% PT coated and TPT coated with different concentrations of trimethylated silica (PT) (The photos in blue and green boxes refer to the water contact angle profiles after laundering and crocking, respectively).

The coating of TPU-PDMS-TMS has brought protection against water to polycotton fabrics. Figure 4 shows the water contact angle of polycotton fabrics before and after coating. Un treated polycotton fabrics have very good wettability to water with an immediate water contact angle of around 50°, and no evident water contact angle can be observed after one minute as the droplet will be absorbed into the fabric. A simple knife coating of TPU has resulted in an enhancement of water contact angle to 134 ± 10° with hysteresis of 4.61 ± 2°, indicating that a hydrophobic surface has been formed on the surface of the fabric. The reason for the hydrophobicity of the surface of the polycotton fabric is the decrease of surface tension. Previous study has found that waterborne polyurethane (WPU) coating decreased the surface tension of cotton fabrics to 72.8 dynes/cm^−1^, resulting in a water contact angle of 94° [21]. TPU coating is more effective in reducing the surface tension than WPU, and it decreases the surface tension to 46.2–39.4 dynes/cm for polycotton fabrics to be resistant to water and liquids/chemicals.

The 8% PT coated polycotton fabric displays a water contact angle of 155 ± 4° with hysteresis of 0.36 ± 1°, and this is due to the further lower surface tension (22.0 dynes/cm) of PT [11,24]. The polydimethylsiloxane groups in PT reduces the surface tension of polycotton fabrics. It is also noticed that the hydrophobicity is probably due to the rough surface of the coated fabric due to the particles on fibers. The water contact angles of TPTs are 139 ± 1° (hysteresis 1.49 ± 1°), 142 ± 7° (hysteresis 0.38 ± 1°), 140 ± 8° (hysteresis 8.01 ± 1°), and 127 ± 10° (hysteresis 9.39 ± 2°) with PT concentrations of 2, 4, 6 and 8%, respectively. The water contact angles of TPTs are slightly higher than that of TPU as shown in Figure 4, suggesting that PT further enhances the hydrophobicity of the TPU on the surface of polycotton fabrics. It is noted that the concentration of PT has little effects on the water contact angle. Compared with the water contact angle of WPU as reported [21], the TPT coating has resulted in an enhanced hydrophobicity evidently as the water contact angle (around 140°) is higher than that of WPT coating (around 127°). For comparison, the water contact angle of the widely-applied Gore-Tex is around 110 °C, while TPT coating brings better hydrophobicity to fabrics with a higher water contact angle.

Durability of coating is always an issue as the linking between the coated components and the substrate is very weak, so it is very hard to maintain the added functions when the coated fabrics are subject to different cycles of laundering or crocking [25,26,27]. The TPU-PDMS-TMS coating shows an excellent durability against laundering and crocking, as indicated in Figure 4. The PT coating shows a reduction of around 14% after laundering and crocking, and this is due to the removal of polydimethylsiloxane particles from the surface of the coated polycotton fabric. Statistical analysis suggests that the water contact angles after laundering and crocking are significantly different to the coated for 8% PT. However, TPU forms a smooth membrane on the surface of fibers and thus the durability against washing and crocking is very high, as shown in Figure 4. Existence of TPU enhances the durability of PT, so that the TPT coatings with different concentrations of PT show reductions in water contact angle of less than 5% after laundering and crocking. PDMS alkyl chains have strong bonding strength between the fabric and the TPT coating, thus the durability of the coating is very high. The statistical analysis shows that the water contact angles are not significantly different for TPU and TPTs, indicating that the hydrophobicity is very durable against laundering and crocking. It is noticed that for WPT coating there is an 8–15% reduction in water contact angle after laundering or crocking the coated fabrics [21], the durability of TPT coating is thus more durable than that of WPT.

#### 3.2.2. Water Repellency

**Figure 5:**
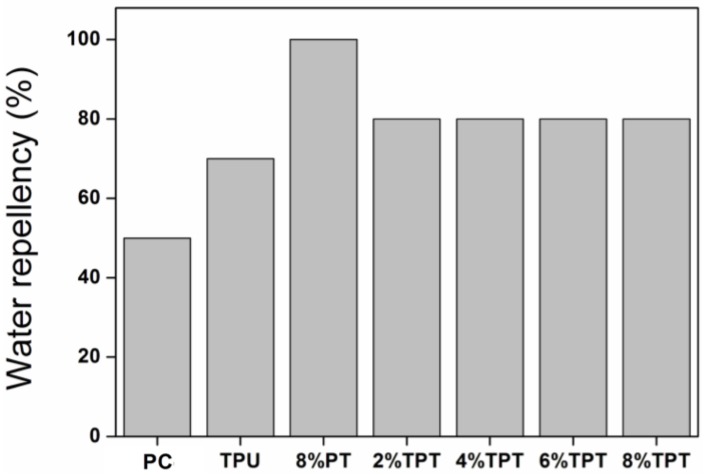
Water repellency of the uncoated polycotton fabric and polycotton fabrics coated with TPU, 8% PT and TPT with different concentrations of PT.

Polycotton fabrics are not waterproof, and the water repellency of the untreated polycotton fabric is as low as 50%. The low water repellency is due to the porous structure of the fabric and the wettability of the cotton fibers in the fabric. Coating of TPU has enhanced the water repellency to 70%, as shown in Figure 5. The surface of polycotton fibers has been covered with TPU membrane after coating (Figure 3b), so that water can only penetrate through the pores and channels within the fibrous structure. It is noted that PT coating brings 100% water repellency to polycotton fabrics. The surface energy of the PT coated polycotton fabric is very low due to the siloxane groups, resulting in a high water contact angle and the absolute water repellency. TPT coated fabrics show the water repellency of 80%, suggesting a 10% enhancement than the TPU coated. However, the water repellency of TPTs is lower than that of 8% PT, and this is due to their relatively lower surface tension. The recrystallization of the alkyl long chains of PDMS leads to stronger bonding strength between the middle chains of TPU, thereby the water repellency of coated fabric has been enhanced [8,21]. The concentration of PT does not affect the water repellency of the TPT coated fabrics, as shown in Figure 5. It is evident that coating with TPU-PDMS-TMS has brought excellent water repellency to polycotton fabrics.

#### 3.2.3. Oil Repellency

**Figure 6:**
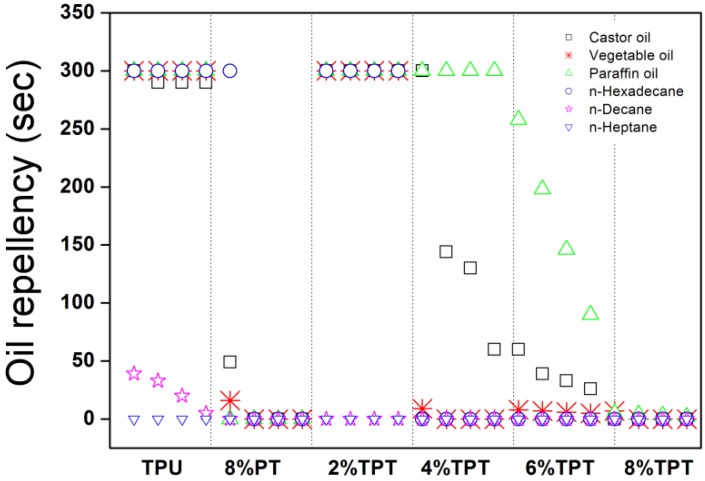
Oil repellency of fabrics coated with TPU, 8% PT and TPTs with different concentrations of PT (For each sample, the four series of data from left to right stand for the oil repellency without tension and with the tension of 1, 3 and 5 NP, respectively).

The coated polycotton fabrics display different oil repellency, as illustrated in Figure 6. The TPU coated fabric shows good repellency to *n*-heptane, paraffin oil and *n*-hexadecane, and this is because that the TPU coating has resulted in a high surface tension to prevent these hydrocarbons oils from penetrating into the coated fabric [20,28]. The surface tension of TPU coated polycotton fabric is similar to *n*-decane (23.0 dynes/cm) and *n*-heptane (19.8 dynes/cm), so that the fabric is less repellent to them. The 8% PT coated polycotton fabric exhibits the poor repellency to *n*-heptane because the *n*-heptane has been used as the diluted solvent to prepare PT solutions. The surface tension of 8% PT (22.0 dynes/cm) is very close to that of *n*-heptane (19.8 dynes/cm) and *n*-decane (23.4 dynes/cm), so that these oils penetrate into the fabric as shown in Figure 6. The droplet of *n*-hexadecane stays on the surface of the fabrics due to its relatively higher surface tension (27.0 dynes/cm) [21].

From Figure 6 it is noticed that the 2% TPT shows excellent oil repellency against the castor oil, paraffin oil, vegetable oil and *n*-hexadecane. These droplets expand on the top layer of PT without penetrating into the underneath TPU layer. The oil repellency declines with the increase of the concentration of PT, and this is due to the resulted lower surface tensions of the top layer that allow the droplets to penetrate into the fabrics. When the coated fabrics are under a tension, the repellency to some oils deteriorates, but in most cases, it remains the same. As shown in Figure 6, the repellency against castor oil drops gradually with the increase of tension for 4% TPT and 6% TPT. Besides, the repellency against paraffin oil for 6% TPT drops evidently with the increase of tension.

The oil repellency remains the same after laundering with only slight declines, but PT coating shows very poor durability against laundering. On the other hand, the oil repellency deteriorates apparently after crocking.

#### 3.2.4. Aqueous Liquid Repellency

**Figure 7:**
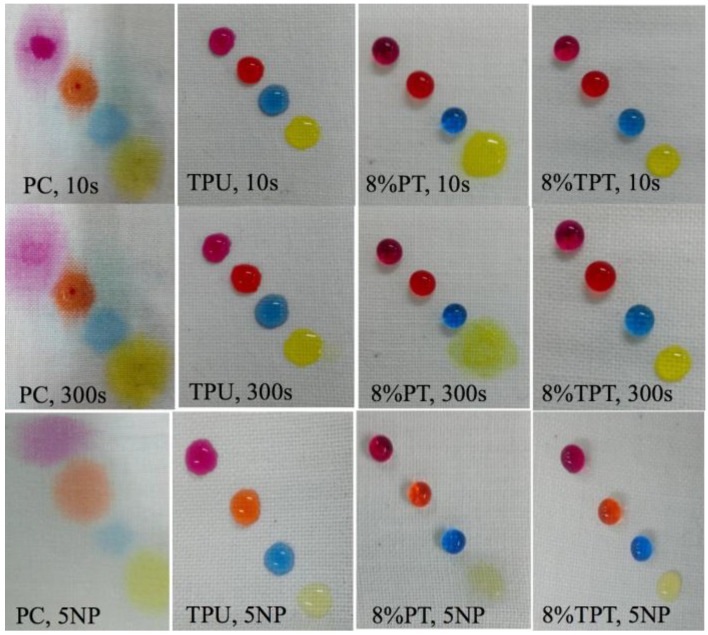
Aqueous liquid repellency of the uncoated polycotton fabric, TPU coated, 8% PT coated and 8% TPT coated fabrics (water/alcohol composition ratios are 98/2 for shopping pink, 90/10 for orange, 80/20 for blue and 60/40 for yellow, respectively).

The uncoated polycotton fabric is not resistant to any aqueous liquids due to its hydrophilic nature, while the TPU coated fabric shows excellent resistance to all the water/alcohol aqueous liquids, as shown in Figure 7. It can be seen that the droplets on the TPU coated fabric are in the shape of semi-sphere.

The 8% PT coated polycotton fabric is repellent to all the aqueous liquids except for the one with the ratio of 60/40. The surface tension of the aqueous liquids decreases from 44 to 24 dynes/cm with the increase of the ratio of alcohol in the mixture. The surface tension of 8% PT is 22.0 dynes/cm, similar to that of the 60/40 aqueous liquid, thus the fabric is not resistant to it [21]. Besides, the droplets on 8% PT are in the shape of a near-sphere, suggesting better repellency than that of TPU coating. The reason for the enhanced repellency for 8% PT is probably due to its superhydrophobicity.

The 8% TPT shows the excellent repellency to all kinds of aqueous liquid, and the repellency is not altered after 300 s, as shown in Figure 7. When the fabrics are subject to an external tension, the aqueous liquid repellency is not changed. It is evident that the aqueous liquid repellency from this coating is very favorable to protective clothing that is usually subject to different tensions.

#### 3.2.5. Chemical Resistance

Chemical resistance depends on the surface tension of the coated fabrics, and usually the fabric is resistant to chemicals with higher surface tensions. On the other hand, if the surface tension of a chemical is closer to the coated fabric, the chemical will penetrate into the fabric within a short period of time. Polycotton fabrics are not resistant to chemicals due to their excellent wettability, as shown in Table 2.

The TPU-coated fabric shows excellent resistance to liquid chemicals, such as acetonitrile, acetic acid, butadiene, dimethylformamide, *n*-heptane, *n*-hexadecane, *iso*-propyl alcohol, paraffin oil and sodium hydroxide. This is due to the higher surface tensions of these chemicals than that of the TPU-coated fabric. TPU coating brings omniphobic surface to the coated fabric with the excellent chemical resistance [1,24]. However, TPU coating is not resistant to *n*-hexane, triethylamine and *n*-decane due to their similar surface tensions.

With its lower surface tension, the 8% PT coated fabric is limitedly resistant to chemicals including acetic acids, butadiene, dimethylformamide, *n*-hexadecane and sodium hydroxide. The TPT coating is resistant to most of chemicals as indicated in Table 2, as the coating combines the resistance from both TPU and PT. Increase of the concentration of PT has limited effects on the chemical resistance. Similar to the oil repellency, the chemical resistance is not severely affected after laundering but it deteriorates evidently after crocking.

It is evident that the TPT coating has brought the excellent chemical resistance to polycotton fabrics, and the versatile protective capacity of the coated polycotton fabrics can be applied in various areas where chemicals are involved.

### 3.3. Comfort

#### 3.3.1. Air Permeability

**Figure 8:**
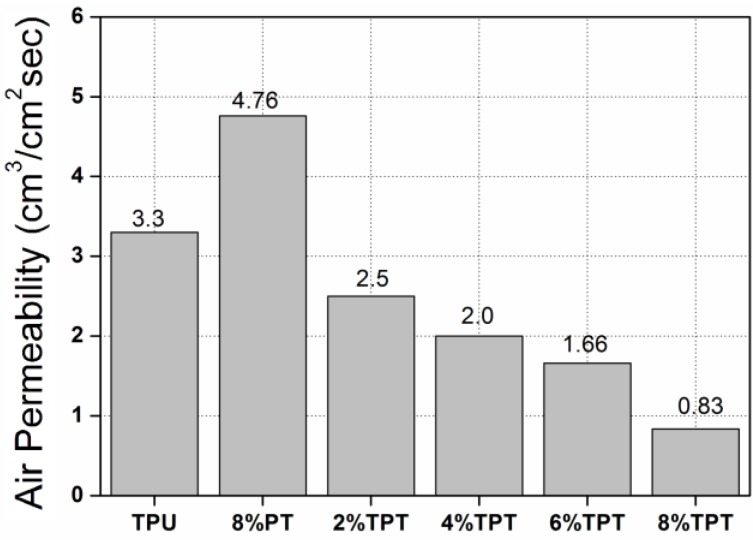
Air permeability of polycotton fabrics coated with TPU, 8% PT, and TPTs with different concentrations of PT.

Air permeability of the untreated polycotton fabric is 95.0 cm^3^/cm^2^/s, indicating that the airflow can easily penetrate through the pores of the fabric. Coating inevitably blocks the pores of fabrics and thus the air permeability of the as-coated fabrics usually deteriorates [9,16,29]. As shown in Figure 8, TPU-coated fabric has an air permeability of 3.3 cm^3^/cm^2^/s and the 8% PT has that of 4.76 cm^3^/cm^2^/s. It is evident that TPU coating has evident effects in blocking the pores of polycotton fabrics than PT. TPT-coated fabrics have even lower permeability than that of TPU, and the permeability decreases further with the increase of the concentration of PT. The adding of PT on the top of TPU would further block the pores between the neighboring fibers, thus the air permeability decreases further with the increase of the concentration of PT.

Nevertheless, the air permeability has been improved by replacing WPU with TPU in the coating [21]. WPT coating shows an air permeability of 0.1–0.2 cm^3^/cm^2^/s, which is much lower than that of TPT coating. WPU forms a uniform membrane on the surface of polycotton fabrics without fibers being seen from SEM photos, while TPU coating does not fully cover the surface of the fabric with fibers observable from SEM photos. TPT coating thus allows air to penetrate through the limitedly preserved pores of the fabrics, resulting in a higher air permeability than WPT coating.

#### 3.3.2. Water Vapor Resistance and Permeability

**Figure 9:**
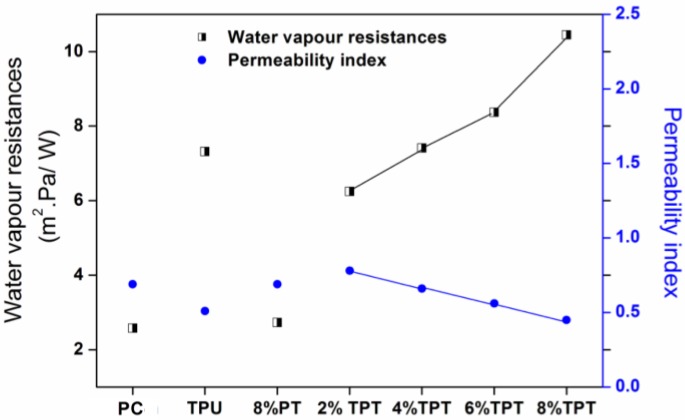
Water vapor resistance and permeability index of the uncoated polycotton fabric, TPU coated, 8% PT coated and TPT coated with different concentrations of PT.

Water vapor resistance is the factor to determine the breathability of fabrics. TPU coating has deteriorated the water vapor permeability of polycotton fabrics; the water vapor resistance and permeability index are shown in Figure 9. This is due to the blocking of the pores of polycotton fabrics by the TPU membrane. The coating of PT doesn’t affect the breathability of polycotton fabrics much as most of pores within fabrics are preserved. The increase of the concentration of PT in TPT coatings has significant effects on the breathability of the coated fabrics, and the higher the concentration of PT, the higher the water vapor resistance [30]. In this case, the increase of the thickness of the coated layers from increasing the PT concentration results in the worse breathability as more pores of the coated fabrics have been blocked. For comparison, Gore-Tex has an excellent breathability, the TPT coating doesn’t exhibit as good breathability as Gore-Tex though its surface is more superomniphobic.

#### 3.3.3. Thermal Resistance

**Figure 10:**
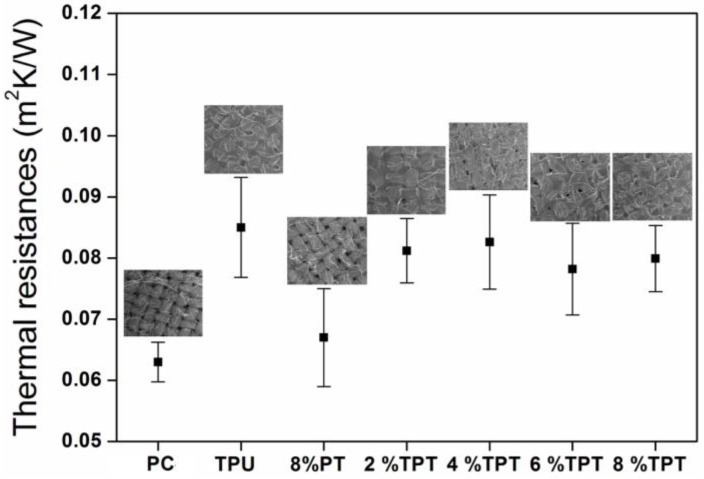
Thermal resistance of the uncoated polycotton fabric, TPU coated, 8% PT coated and TPT coated with different concentrations of PT.

The thermal resistance of polycotton fabrics is enhanced after coating, as shown in Figure 10. The uncoated polycotton fabrics have open pores (as shown the inset) to allow heat exchange between the hotplate and the environment, so that the thermal resistance is very low. The PT coated polycotton fabric has a similar fibrous structure as indicated in the inserted SEM photo, but the thickness of the coated fabric is higher than the uncoated. The thermal resistance of PT coated polycotton fabric is thus slightly higher than that of uncoated [31], but the data of thermal resistance is not significantly different according the standard deviations in Figure 10. TPU coating exhibits a thermal resistance of 0.085 m^2^ K/W, which is much higher than that of the uncoated polycotton fabric (0.063 m^2^ K/W) with a statistically significant difference. The enhancement of thermal resistance after coating of TPU is due to the blocking of the open pores, as shown in the inserted SEM photo. The blocked pores capture static air within the fibrous structure, resulting in less heat transfer with a higher thermal resistance. The thermal resistance of TPTs is similar to that of TPU, with a significant difference to the untreated cotton fabrics. The increase of PT concentration in TPT coating has limited effects on the thermal resistance, as seen from Figure 10. In this case, the enhancement of thermal resistance is mainly from the blocking of open pores within the fibrous structure, whereas the increase of thickness of the coating dose not contribute much to the increase of thermal resistance.

#### 3.3.4. Moisture Management Property

**Figure 11:**
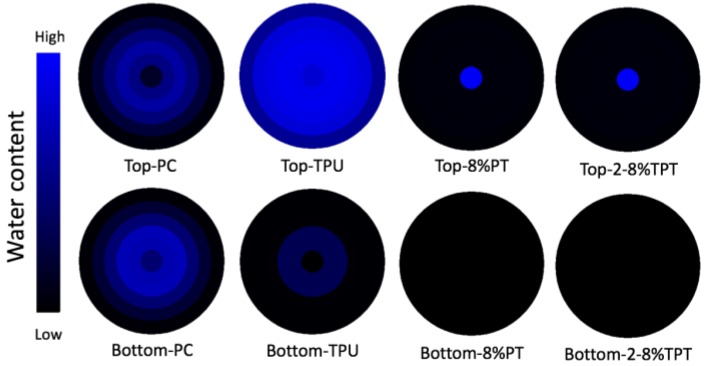
Moisture management tester (MMT) profile of the uncoated polycotton fabric, TPU coated, 8% PT coated and TPT coated with different concentrations of PT.

The moisture management property of polycotton fabrics has changed after the coating, as indicated by the MMT profiles in Figure 11. For polycotton fabrics, water can transport across the fibrous structure easily due to its excellent wettability and wicking property [32,33]. There are no evident differences between the top and bottom surfaces for polycotton fabrics in terms of the MMT index, as shown in Table 3. Coating of TPU has made the top surface of the polycotton fabric smooth for water to spread, as shown the MMT profile with a larger wet area. However, a smaller amount of water has been transported to the bottom surface, so that the one-way transport capability is significantly lower. It is evident that TPU coating has enhanced the water spreading performance on the top surface but deteriorated the water transport capability of the polycotton fabrics. The overall moisture management capacity (OMMC) has changed from 0.47 to 0.04 after TPU coating.

The water droplet on 8% PT appears as a sphere, as shown in Figure 11. The rough surface and low surface energy of PT have made the polycotton fabric surface superhydrophobic, thus the water does not spread on the top surface nor does it transport to the bottom side. The bottom surface has not been wetted, as shown the profile in Figure 11. It is evident that PT coating has made the polycotton fabrics superhydrophobic and water cannot penetrate through the fabrics. Besides, TPT coatings have the similar moisture management property with PT coating, and this is due to the existence of PT on the surface from these coatings.

## 4. Conclusions

A soft, flexible, highly durable, comfortable, and versatile protective coating for polycotton fabrics was developed by the pad-knife-pad coating of TPU-PDMS-TMS. The coating brought a thin layer to the surface of fibers while the fibrous structure of the fabric was not severely affected. The active TPU combined with PDMS-TMS to form a crosslinked network, providing flexible and soft segments with low surface energy siloxane groups to the polycotton fabrics. The coated polycotton fabrics showed a superhydrophobic surface with a water contact angle of 142–155°, and the superhydrophobicity was durable against different cycles of laundering and crocking. The enhanced durability of the samples has been achieved through the recrystallization of the long chains of the methyl groups of the PDMS and Si–OCH_3_ bonding imparted between the substrate and the TPT coating. The coating also showed the excellent repellency against water, oil, aqueous liquids and different chemicals. The versatile protection was not noticeably affected when the fabric was subject to tensions. The air permeability together with the water vapor permeability of the polycotton fabrics deteriorated after coating due to the blocking of pores by the deposited TPT. The thermal resistance of the coated polycotton fabrics was higher than that of the uncoated polycotton fabric due to the trapping of static air within the fabric structure by the coated layers. TPU coating enhanced the water spreading capacity on the surface of polycotton fabric but deteriorated the water transport capacity. The TPT coated fabrics exhibited water droplets on their surface and showed poor moisture management capacity.

## Figures and Tables

**Table 1:** Detected Functional groups for all coated and uncoated polycotton fabrics by FTIR.

Samples	Functional groups	Wavenumber (cm^−1^)	Attributed to
Polycotton	O–H	3382 (ѵ)	Stretching of hydroxyl group
	CH_2_	2970 (ѵs)	Stretching of methyl group
	CH	1425 (δ)	Bending of methyl group
	C–O–C	1315 (a)	Asymmetric stretching of ester group
	C–O	1017 (a)	Asymmetric stretching of carbon monoxide group
TPU	NH	3335 (ѵ)	Stretching of amide group
	CH_2_	2955 (ѵa)	Symmetric stretching of methyl group
	C=O	1728	Stretching of carbonyl group
	C=C Benzene ring	1597–1414 (δs)	Bending of symmetric of carbon with double bonding in ring
PDMS-TMS	CH_2_	2975 (a)	Asymmetric stretching of methyl group
	Si–CH_3_	1270–1250 (ѵa)	Asymmetric stretching of methyl and silicone group
	Si–(CH_3_)_n_	870–700 (δ)	Bending stretching of silicone and no methyl groups
	Si–O–Si	1059–1020 (ѵa)	Asymmetric stretching of silicones and oxygen group
TPU-PDMS-TMS	NHCOO–Si–O–CH_3_	1713–749	Crosslinking between TPU chain and PDMS-TMS groups

Note: ѵ: stretching, δ: bending, ρ: rocking, a: asymmetric and s: symmetric.

**Table 2:** Chemical resistance(s) of uncoated and coated polycotton fabrics.

Chemicals	Surface tension (dynes/cm)	Polycotton	TPU	8% PT	2% TPT	4% TPT	6% TPT	8% TPT
Triethylamine	19.70	0	0	0	0	0	0	0
Tetrahydrofuran	26.40	0	62	0	82	91	141	167
*n*-Hexane	18.43	0	0	0	0	0	0	0
Dichloromethane	26.80	0	70	5	0	16	30	35
Acetone	23.20	0	70	10	5	16	30	57
Acetonitrile	28.70	0	186	0	114	228	289	297
Methanol	22.10	0	146	0	69	75	85	137
Toluene	28.40	0	185	0	0	0	0	0
Dimethylformamide	36.70	0	300	300	300	300	300	300
Acetic acids	27.00	0	300	300	300	300	300	300
Sodium hydroxide	101.04	0	300	300	300	300	300	300
Butadiene	47.01	0	300	300	300	31	16	10
Paraffin oil	26.00	0	300	16	300	45	50	57
*n*-Hexadecane	25.30	0	300	300	118	30	29	28
*n*-Decane	23.80	0	40	0	13	6	5	4
*n*-heptane	20.10	0	300	0	300	300	300	300
*iso*-propyl alcohol	23.00	10	300	10	300	300	300	10
Sulphuric acids	84.00	0	300	300	300	300	300	0

**Table 3:** Moisture management properties of the uncoated polycotton fabric, TPU coated, 8% PT coated and TPT coated with different concentrations of PT (measure time = 120.00 s).

MMT index	Polycotton		TPU		8% PT		2–8% TPT	
	Top surface	Bottom surface	Top surface	Bottom surface	Top surface	Bottom surface	Top surface	Bottom surface
Wetting time (s)	5.90	5.99	4.68	12.23	8.42	120.00	7.58–9.54	120.00
Absorption rate (&/s)	5.73	15.93	67.98	4.89	253.66	0.00	90.20–406.33	0.00
Maximum wetted radius (mm)	15.00	15.00	25.00	0.00	5.00	0.00	5.00	0.00
Spreading speed (mm/s)	2.47	3.20	4.84	0.00	0.58	0.00	0.51–0.64	0.00
One way transport capability	62.08		−624.90		−995.77		−988.00–−1086.26	
OMMC	0.47		0.04		0		0	
